# The NO-cGMP-PKG Axis in HFpEF: From Pathological Mechanisms to Potential Therapies

**DOI:** 10.14336/AD.2022.0523

**Published:** 2023-02-01

**Authors:** Zhulan Cai, Cencen Wu, Yuan Xu, Jiageng Cai, Menglin Zhao, Lingyun Zu

**Affiliations:** ^1^Department of Cardiology, Peking University Third Hospital, Beijing 100191, China.; ^2^Department of Cardiology and Institute of Vascular Medicine, Peking University Third Hospital, China.; ^3^Key Laboratory of Cardiovascular Molecular Biology and Regulatory Peptides, Ministry of Health, China.; ^4^Beijing Key Laboratory of Cardiovascular Receptors Research, Beijing 100191, China

**Keywords:** HFpEF, NO-cGMP-PKG axis, pathological mechanisms, potential therapies

## Abstract

Heart failure with preserved ejection fraction (HFpEF) accounts for almost half of all heart failure (HF) cases worldwide. Unfortunately, its incidence is expected to continue to rise, and effective therapy to improve clinical outcomes is lacking. Numerous efforts currently directed towards the pathophysiology of human HFpEF are uncovering signal transduction pathways and novel therapeutic targets. The nitric oxide-cyclic guanosine phosphate-protein kinase G (NO-cGMP-PKG) axis has been described as an important regulator of cardiac function. Suppression of the NO-cGMP-PKG signalling pathway is involved in the progression of HFpEF. Therefore, the NO-cGMP-PKG signalling pathway is a potential therapeutic target for HFpEF. In this review, we aim to explore the mechanism of NO-cGMP-PKG in the progression of HFpEF and to summarize potential therapeutic drugs that target this signalling pathway.

## 1. Introduction

In the past decade, heart failure with preserved ejection fraction (HFpEF) with left ventricular ejection fraction (LVEF) ≥50% has become more prevalent, accounting for approximately 50% of all cases of heart failure (HF) [1]. The growing prevalence of risk factors and/or common comorbidities, such as population ageing, obesity, and diabetes, may contribute to a continuous increase in the HFpEF incidence [2, 3]. HFpEF is mainly characterized as cardiac structural and functional abnormalities that result in diastolic dysfunction and impaired ventricular filling [4, 5]. Recent preclinical and clinical studies have clearly shown that HFpEF represents a group of heterogeneous pathophysiological clusters with a collection of clinical manifestations involving multiple organ systems and concomitant ageing, rather than a single homogeneous disease [6, 7]. Hypotheses about the pathogenesis of HFpEF are complex and diverse. Myocardial fibrosis, cardiomyocyte energy metabolism disorder, epicardial adipose tissue accumulation, haemodynamic disturbance, hyperactivation of the neuroendocrine system, microcirculatory dysfunction, and the systemic inflammatory response are thought to be involved in the development of the disease [6, 8]. Regrettably, a suitable preclinical animal model that precisely replicates the clinical multiorgan system complications of HFpEF patients is lacking, limiting our understanding of the pathophysiological mechanisms underlying HFpEF [9].

There is a fair amount of evidence demonstrating that the nitric oxide (NO) signalling pathway is disturbed in HFpEF patients, suggesting that it is, at least partly, the cause of cardiac dysfunction. Here, we compiled studies on the potential role of the nitric oxide-cyclic guanosine phosphate-protein kinase G (NO-cGMP-PKG) pathway in the occurrence and development of HFpEF to partially explain the pathogenesis of HFpEF. Additionally, unlike heart failure with reduced ejection fraction (HFrEF), which has effective and definitive interventions, there are limited therapeutic agents that can effectively improve clinical outcomes in patients with HFpEF [10-12]. Due to its growing prevalence and phenotypic diversity, a number of large randomized clinical trials are being carried out to improve the prognosis of patients with HFpEF [13]. Here, we present clinical pharmaceutical strategies targeting the NO-cGMP-PKG pathway for the prevention and treatment of HFpEF, with special attention to progress in HFpEF research and its limitations, providing new insights and theoretical foundations for future HFpEF therapeutic approaches.

## 2.NO-cGMP-PKG signalling pathway in the cardiovascular system

NO can be synthesized in vivo and is a crucial physiological transmitter that functions as an intercellular and intracellular messenger. As the main effector molecule that modulates vascular tone and maintains vascular integrity, NO is mainly produced and released by vascular endothelial cells [14]. Under the stimulation of physical (e.g., shear stress) or chemical (e.g., cytokines and platelet-derived factors) factors, it is synthesized by endothelial NO synthase (eNOS) from its precursor, L-arginine [15]. In vascular endothelial cells, NO inhibits the expression of leukocyte adhesion molecules to reduce vascular inflammation. Additionally, NO mobilizes stem and progenitor cells to maintain vascular homeostasis and facilitate vascular repair under certain pathological conditions [16]. Through extravascular diffusion into vascular smooth muscle cells, NO can bind to its receptor and facilitate the activity of the sGC-cGMP-PKG pathway, causing vascular smooth muscle relaxation and vasodilation [17]. NO may also diffuse to cardiomyocytes that are adjacent to coronary capillaries and endocardial endothelial cells. In the myocardium, the activated NO-cGMP-PKG pathway usually has antihypertrophic, antifibrotic, and angiogenic effects, thus inhibiting cardiac remodelling [18-20]. Under normal physiological conditions, organisms produce an appropriate amount of NO to maintain homeostasis. In certain pathological states (such as atherosclerosis, hypertension and HF), vascular endothelial cells become damaged, which reduces NO production and disrupts the homeostatic balance, thereby aggravating the progression of cardiovascular disease [21-23].

cGMP, a universal intracellular second messenger, is widely distributed in various tissues. Guanylate cyclase (GC) catalyses the transition of guanosine triphosphate (GTP) to cGMP, and phosphodiesterases (PDEs) catalyse the degradation of cGMP [24, 25]. cGMP exerts its function by binding to its intracellular receptors, translating NO signalling and stimulating PKG to induce changes in phosphorylation [26, 27]. Enhancing the synthesis of cGMP or blocking its degradation by PDEs is a viable option for protection against cardiovascular disease [20].

PKG, also known as cGMP-dependent protein kinase, is a serine/threonine protein kinase that is widely expressed in eukaryotic cells and is considered to be the most important downstream target of cGMP [28]. By regulating multiple molecular signal transduction pathways, PKG elicits vasodilation in vascular smooth muscle cells. Moreover, PKG has been shown to activate myosin light chain phosphatase to induce the dephosphorylation of the myosin light chain, relaxing vascular smooth muscle [29]. PKG also inactivates transforming protein RhoA signalling through phosphorylation at Ser188, which suppresses hypertrophic and fibrotic activation of vascular smooth muscle cells [30, 31]. Additionally, PKG generates p-PKG through autophosphorylation, inducing the phos-phorylation of vasodilator-stimulated phosphoprotein, which ultimately contributes to vasodilation and inhibition of platelet aggregation [32, 33].

NO exerts its physiological functions through the cGMP-independent pathway and the cGMP-dependent pathway that is mediated by activation of sGC [28]. Catalysis of sCG induces an increase in the intracellular concentrations of cGMP and its downstream effector PKG, which underlies their diverse biological functions, such as mediation of vascular smooth muscle cell relaxation, inhibition of platelet aggregation, and participation in neurotransmission [34, 35].

## 3.NO-cGMP-PKG signalling pathway in HFpEF

### 3.1 Impaired NO-cGMP-PKG axis in HFpEF

A retrospective clinical study performed transcatheter left ventricular myocardial biopsy in patients with HFpEF to observe PKG activity and cGMP concentration. The cGMP content and PKG bioactivity were found to be significantly reduced in myocardial homogenates from the HFpEF patients [36]. Thereafter, Constantijn Franssen *et al.* [37] observed uncoupling of eNOS and reductions in cGMP content and PKG bioactivity in endocardial biopsies of patients with HFpEF. Moreover, they also detected a deficiency of the NO-cGMP-PKG pathway in obese Zucker spontaneously hypertensive diabetic fatty (ZSF1)-HFpEF rats [37]. In another HFpEF mouse model established by injection of N-nitro-L-arginine methyl ester (L-NAME) combined with a 14-week high-fat diet, levels of NO metabolites in the myocardium decreased, cGMP levels in plasma were reduced, and PKG protein expression was downregulated, indicating that the NO-cGMP-PKG pathway was inhibited in HFpEF [38]. The suppression of the NO-cGMP-PKG pathway was also confirmed in animal models of other species. For example, the aortic NO-cGMP-PKG signalling pathway was observed to be inhibited in HFpEF pig models that were established by injection of deoxycorticosterone acetate and angiotensin II (Ang II) combined with a Western diet for 18 weeks [39].

The downregulation of the NO-cGMP-PKG pathway may be attributed to the reduction in myocardial NO bioavailability caused by the activation of coronary microvascular endothelial inflammation and nitrosation/oxidative stress [40]. Common comorbidities, including obesity [41-43], diabetes [44], hypertension [45], chronic obstructive pulmonary disease (COPD) [46], and chronic kidney disease (CKD) [47], have been postulated to drive the systemic inflammatory state, as shown by elevated levels of circulating cytokines, including IL-1β, IL-6, CRP [48], and TNFα [49, 50]. Sustained systemic inflammation ultimately triggers the activation of endothelial cells within the myocardial microcirculation, promoting the upregulation of adhesion molecules on the surfaces of endothelial cells [37, 51]. The emergence of these homing molecules induces the activation of leukocytes and fosters their mobilization and subendothelial migration to inflamed or injured heart tissue [52, 53].

**Figure 1.**
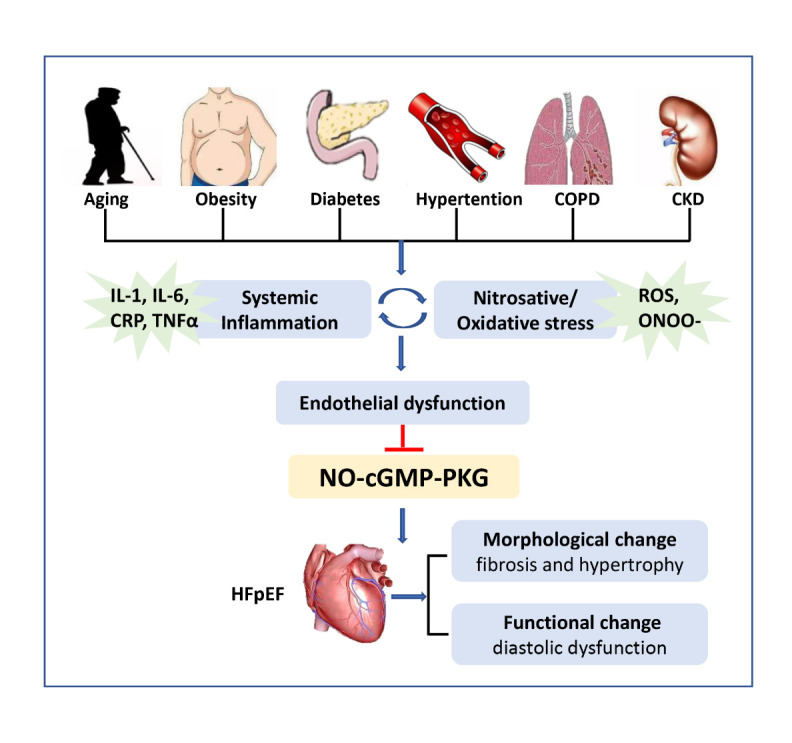
Impaired NO-cGMP-PKG axis in HFpEF. Risk factors for ageing and common comorbidities (including obesity, diabetes, hypertension, chronic obstructive pulmonary disease, chronic kidney disease) drive systemic inflammation, which further activates coronary microvascular endothelial inflammation and nitrosation/oxidative stress. The vicious cycle between inflammation and nitrosation/oxidative stress contributes to endothelial dysfunction, which further disrupts the signalling communication between endothelial cells and other types of cells in the heart. The suppression of NO-cGMP-PKG signalling in HFpEF ultimately results in cardiac structural changes and functional damage. COPD: chronic obstructive pulmonary disease, CKD: chronic kidney disease, IL-1: interleukin-1, IL-6: interleukin-6, CRP: C-reactive protein, TNFα: tumour necrosis factor α, ROS: reactive oxygen species, ONOO-: peroxynitrite, NO: nitric oxide, cGMP: cyclic guanosine monophosphate, PKG: protein kinase G, HFpEF: heart failure with preserved ejection fraction.

Excessive amounts of proinflammatory cytokines further give rise to endothelial production of ROS via NADPH oxidase activation [54], which may explain the high nitrosative/oxidative stress, as supported by left ventricle (LV) biopsy samples from HFpEF patients and ZSF1-HFpEF rats [37]. High nitrotyrosine expression also indicates the low bioavailability of NO in HFpEF myocardium since the rapid interaction between NO and superoxide anions is subsequently followed by the formation of cytotoxic peroxynitrite (ONOO-). In cardiomyocytes, NO is unable to stimulate sGC because of the reduction in its bioavailability, thus lowering the cGMP concentration and decreasing PKG activity. On the other hand, nitrosative stress elicits protein modification, such as S-nitrosylation of cysteine residues, and disrupts protein function [55]. For example, nitrosative stress induces S-nitrosylation of the endonuclease inositol-requiring protein 1α (IRE1α), ultimately resulting in defective production of X-box binding protein 1 (Xbp1s) in cardiomyocytes [40]. The deregulation of IRE1α-Xbp1s is responsible for cardiac dysfunction in HFpEF [40].

The suppression of NO-cGMP-PKG signalling has been observed in clinical patient samples and a variety of experimental animal HFpEF models, which encourages investigators to pursue more targeted therapy strategies involving rescue of the NO-cGMP-PKG signalling pathway. Regarding HFpEF treatment strategies, over the last few years, many research institutions and personnel have made numerous efforts to identify drugs that act on this signalling pathway, and continuous efforts, which we will discuss below, are ongoing.

### 3.2 Pathophysiological actions of the NO-cGMP-PKG axis in HFpEF

Coronary microvascular endothelial inflammation and oxidative stress trigger endothelial dysfunction, which results in reduced NO signalling and affects neighbouring cardiomyocytes and cardiac fibroblasts via the sGC-cGMP-PKG signalling pathway [56-58]. Decreased NO bioavailability and reduced myocardial PKG content eventually lead to structural and functional cardiac changes, contributing to the development of HFpEF [36]. Structurally, this maladaptation of the HFpEF heart is characterized by interstitial fibrosis along with macroscopic and microscopic hypertrophy [53, 59]. With respect to function, diastolic dysfunction classically manifests as both delayed myocardial relaxation and a loss of compliance owing to increased cardiomyocyte stiffness [60, 61] (Fig. 1).

The impact of NO-cGMP-PKG signalling on cardiac hypertrophy and fibrosis has been identified in various preclinical experiments and clinical trials. NO exerts a direct antifibrotic and antihypertrophic effect on cardiovascular homeostasis by antagonizing endothelin-1, angiotensin II, and aldosterone. Low bioavailability of NO allows these molecules to exert their profibrotic effects and favours the proliferation of fibroblasts and myofibroblasts [62, 63]. In cultured neonatal rat cardiomyocytes, NO or cGMP analogues were shown to reverse the hypertrophic response induced by norepinephrine [62]. PKG has been suggested to be involved in the regulation of cardiovascular disease. In particular, it negatively regulates myocardial hypertrophy and remodelling in the cardiovascular system [64, 65]. Fiedler *et al.* [66-68] first confirmed that the antihypertrophic response of PKG in cultured cardiomyocytes is mediated by inactivation of the calcineurin-NFAT signalling pathway via inhibition of the L-type Ca^2+^ channel current. However, mice with cardiomyocyte-restricted PGKI deletion were susceptible to dilated cardiomyopathy with adverse cardiac remodelling when subjected to angiotensin II injection or transverse aortic constriction [69]. In mice with pressure overload-induced cardiac hypertrophy, sildenafil inhibited the decomposition of cGMP by PDE5 to improve myocardial PKG bioactivity, thus suppressing cardiac hypertrophy and interstitial fibrosis via the inactivation of the prohypertrophic signalling pathways [19, 70].

Insufficient NO-cGMP-PKG signalling between endothelial cells and cardiomyocytes affects myocardial relaxation and myocardial stiffness in addition to the regulation of cardiac hypertrophy and fibrosis.

Low endomyocardial NO bioavailability attributed to reduce NO concentrations or increased superoxide production can impair LV distensibility and contribute to diastolic dysfunction [71]. The improvement in diastolic LV stiffness in patients with diastolic dysfunction due to coronary infusions of NO donors [72] is consistent with the results of an experimental study in which administration of an eNOS enhancer could attenuate LV diastolic dysfunction in a rat HFpEF model [73].

The sarcomeric protein titin, which functions as a molecular spring, affects resting tension and passive stiffness in cardiomyocytes. Titin compliance is attributed to posttranscriptional changes and posttranslational modifications, including isoform expression and phosphorylation [74]. Alternative splicing gives rise to two main myocardial titin isoforms: the stiff N2B isoform and the more compliant N2BA isoform. Decreased phosphorylation of the stiff N2B titin has been proven by myocardial biopsy from HFpEF patients [75], which is consistent with preclinical models, and the hypophosphorylated N2B titin isoform is thought to be responsible for the enhanced passive stiffness of cardiomyocytes [76, 77]. Low PKG activity promotes elevated human myocardial passive stiffness by hypophosphorylation of the N2B titin isoform [78, 79], which can be rescued by in vitro treatment with PKG [36]. Short-term cGMP enhancement by sildenafil treatment in aged hypertensive dogs improved LV diastolic distensibility partly via the restoration of N2B titin phosphorylation [80]. The above reasons could, at least in part, explain why individual cardiomyocytes and cardiac muscle strips isolated from HFpEF patients develop increased diastolic tension or have higher F(passive) values [81, 82].

## 4. Therapeutic strategies targeting the NO-cGMP PKG signalling pathway

Significant progress has been made in pharmacological interventions against HFrEF in recent years. However, confirmation of the criteria for the diagnosis and management of HFpEF has been challenging because of the heterogeneity of the clinical manifestations and the complexity and diversity of the underlying pathophysiological mechanisms. Current treatment options for HFpEF patients mainly involve comprehensive strategies, such as amelioration of the patients’ symptoms, management of existing cardiovascular diseases and comorbidities, and early intervention of cardiovascular disease risk factors. Experimental data on the benefits of pharmacological therapy in HFpEF have indicated that targeting the NO-cGMP-PKG axis may serve as a promising therapeutic strategy in interventions for HFpEF. Preclinical evidence indicates that perturbation of this signal transduction cascade is a specific pathological mechanism that underlies HFpEF and is partly responsible for myocardial fibrosis and cardiomyocyte stiffness, eventually driving diastolic dysfunction.

**Figure 2.**
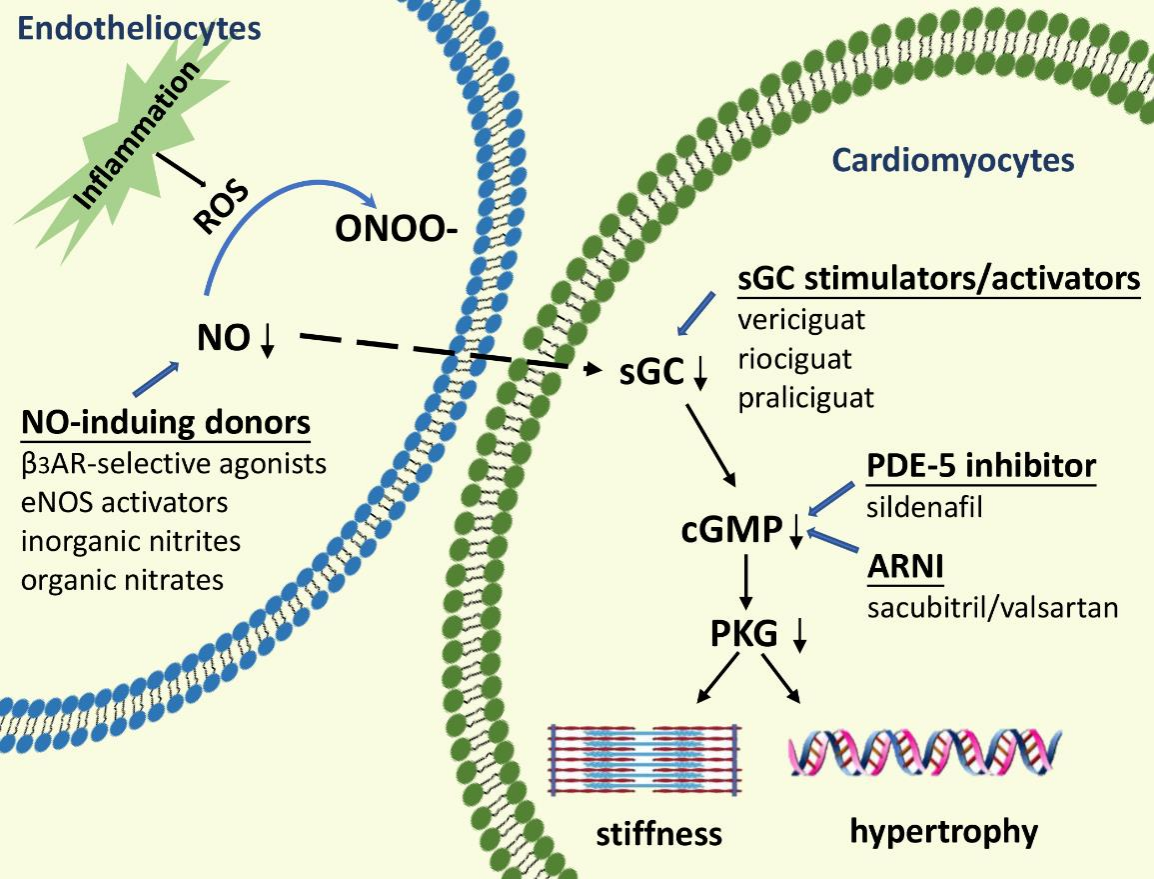
Targeting NO-cGMP-PKG signalling with pharmacological strategies. Endothelial inflammation triggers nitrosation/oxidative stress, and the limited biological activity of NO due to the reaction with ROS to generate ONOO^-^ fails to activate the sGC-cGMP-PKG axis. Lack of PKG activity is linked to increased passive stiffness and hypertrophy of cardiomyocytes. Low NO bioavailability could be alleviated by NO-inducing drugs, including β_3_AR selective agonists, eNOS enhancers, inorganic nitrites/nitrates, or organic nitrates. sGC can be restored by administration of sGC stimulators/activators such as vericiguat, riociguat, and praliciguat. PDE5is and ARNIs predominantly rescue the downstream signalling pathway by increasing the concentration of cGMP. ROS: reactive oxygen species, ONOO^-^: peroxynitrite, NO: nitric oxide, sGC: soluble guanylate cyclase, cGMP: cyclic guanosine monophosphate, PKG: protein kinase G, β_3_AR: β_3_ adrenergic receptor, eNOS: endothelial nitric oxide synthase, PDE5is: phosphodiesterase-5 inhibitors, ARNIs: angiotensin receptor neprilysin inhibitors

Therefore, intervention in the NO-cGMP-PKG signalling pathway with sGC activators/stimulators, phosphodiesterase-5 inhibitors (PDE5is), angiotensin receptor neprilysin inhibitors (ARNIs), sodium-dependent glucose transporters 2 inhibitors (SGLT2is) and NO-inducing drugs, including β_3_ adrenergic receptor (β_3_AR)-selective agonists or eNOS enhancers, organic nitrates and inorganic nitrites/nitrates, has been attempted (Fig. 2) (Table 1).

**Table 1:** Currently published clinical trials of regulation of NO-cGMP-PKG axis in HFpEF.

study name	year	intervention	setting	study size	identifier ID	primary outcome	ref.
*Inorganic nitrates/nitrites*							
NEAT-HFpEF	2015	isosorbide mononitrate	EF≥50%	110	NCT02053493	no change in QOL or 6-MWD	83
Acute Effects of Inorganic Nitrite on Cardiovascular Hemodynamics in HFpEF	2015	inorganic nitrite infusion	EF≥50%	28	NCT01932606	reduce PCWP during exercise	86
Effect of Inorganic Nitrates on Arterial Hemodynamics and Exercise Capacity	2015	dietary inorganic nitrate	EF≥50%	17	NCT01919177	improve submaximal aerobic endurance	88, 89
Pharmacokinetics, Pharmacodynamics, and Impact of Inorganic Nitrate on Exercise in HFpEF	2017	oral inorganic nitrate	EF>50%	12	NCT02256345	improve exercise duration and QOL	90
INDIE-HFpEF	2018	inhaled inorganic nitrite	EF≥50%	105	NCT02742129	no effect in peak VO_2_, and the NYHA classification, NT-proBNP, E/E' and QOL	92
Inhaled Sodium Nitrite on HFpEF Fraction	2016	inhaled inorganic nitrite	EF≥50%	26	NCT02262078	reduce ventricular filling pressure and PAP	94
*Soluble guanylyl cyclase stimulators and activators*							
DILATE-1	2014	riociguat	EF>50%	39	NCT01172756	improvement of diastolic function	95
SOCRATES-PRESERVED	2017	vericiguat	EF≥45%	477	NCT01951638	improve QOL	96, 97
VITALITY-HFpEF	2020	vericiguat	EF≥45%	789	NCT03547583	No change in QOL	98
CAPACITY-HFpEF	2020	praliciguat	EF≥40%	196	NCT03254485	No change in peak VO_2_	99
*Phosphodiesterase-5 inhibitors*							
PDE5i and PH in Diastolic Heart Failure	2011	sildenafil	EF>50%	44	NCT01156636	improved pulmonary hemodynamics	102
Sildenafil in HFpEF and PH	2015	sildenafil	EF≥45%	52	NCT01726049	no change in QOL	104, 105
RELAX	2013	sildenafil	EF≥50%	216	NCT00763867	no change in QOL or 6-MWD	106
*Angiotensin receptor neprilysin inhibiton*							
PARAMOUNT	2012	sacubitril- valsartan	EF≥45%	307	NCT00887588	reduced NT-proBNP level and improved NYHA classification	109
PARAGON-HF	2019	sacubitril- valsartan	EF≥45%	4822	NCT01920711	no change in HF hospitalizations and cardiovascular death	110
*Sodium-dependent glucose transporters 2 inhibitor*							
SOLOIST-WHF	2021	sotagliflozin	--	1222	NCT03521934	reduce cardiovascular death and hospitalization for HF	120
EMPEROR-Preserved	2021	empagliflozin	EF>40 %	5988	NCT03057951	reduce cardiovascular death and hospitalization for HF, improve QOL	11, 121
CHIEF-HF trial	2022	canagliflozin	--	476	NCT04252287	improve QOL	122
PRESERVED-HF	2021	dapagliflozin	EF≥ 45%	324	NCT03030235	improve QOL or 6-MWD	12

QOL: quality of life, 6-MWD: 6-min walking distance, PCWP: pulmonary capillary wedge pressure, VO_2_: oxygen consumption, NYHA classification: New York Heart Association classification, PAP: pulmonary artery pressure, NT-proBNP: N-terminal pro B-type natriuretic peptide, PH: pulmonary hypertension, HF: heart failure

### 4.1 NO-donating drugs

Considering the impairments in the NO signalling cascade in cardiomyocytes owing to disturbed paracrine signalling between coronary microvascular endothelial cells and neighbouring cardiomyocytes, NO donors were presumed to restore the myocardial NO concentration in the HFpEF population.

However, it is not recommended to directly administer NO donors such as organic nitrates (isosorbide) to patients with HFpEF. In the Neat-HFpEF trial with 110 HFpEF patients, the effect of isosorbide mononitrate on the activity tolerance of HFpEF patients was evaluated. Treatment with isosorbide mononitrate (120 mg/d) for 6 weeks did not improve quality of life (QOL) or submaximal exercise capacity and even led to a decrease in activity level [83]. A possible reason for these unexpected outcomes is that isosorbide mononitrate potentially suppresses cardiac output due to its strong vasodilatation effect, which significantly attenuates systemic blood pressure.

On the other hand, inorganic nitrate, which is considered an important NO reservoir in vivo, is an important alternative agent for rescuing NO signalling in HFpEF and seems to improve ventricular performance under stress, especially during exercise [84, 85].

A haemodynamic trial randomly divided 28 HFpEF patients into an acute sodium nitrite infusion group and a matching placebo infusion group, showing that sodium nitrate could lower pulmonary capillary wedge pressure (PCWP) during exercise, which was related to the improvement of cardiac output reserve with exercise [86]. Inorganic nitrite precisely exerts its unique influence on haemodynamics during exercise, presumably when patients benefit the most from vasodilation [87]. A crossover study found that inorganic nitrate treatment in the form of beetroot juice for 1 week can improve the submaximal aerobic endurance in patients with HFpEF [88, 89]. A subsequent study also showed that HFpEF patients given oral inorganic nitrate (potassium nitrate) for 2 weeks had a better exercise duration and QOL [90]. Apart from the beneficial effects on arterial wave reflection, the positive results are possibly supported by the restoration of myocardial NO and peripheral vasodilation. Similarly, HFpEF subjects who received sodium nitrite therapy exhibited improved arterial compliance and elastance during exercise and reduced aortic wave reflections at rest, indicating that inorganic nitrite has a beneficial effect in mitigating arterial stiffening with exercise [91].

In contrast to the above studies of favourable impacts of organic nitrates on HFpEF, a multicentre crossover trial showed that inorganic nitrate, administered via aerosol inhalation for 4 weeks, failed to improve the New York Heart Association (NYHA) functional classification, N-terminal pro-B-type natriuretic peptide (NT-proBNP) level, diastolic function (E/E'), peak oxygen consumption (VO_2_), or QOL [92]. Sujimoto and Kajio [93] conducted a mean 3.1-year follow-up of HFpEF patients treated with inorganic nitrate and found that the risk of cardiovascular death among patients taking inorganic nitrite was significantly higher than that among those who did not take nitrate, meaning that long-term administration of inorganic nitrite in patients with HFpEF was not beneficial and that the use of nitrate was related to a significantly increased risk of adverse cardiovascular events among patients with HFpEF. However, acute inhalation of nitrite seemed to benefit HFpEF patients by reducing ventricular filling pressure and pulmonary artery pressure (PAP) during both rest and exercise [94].

Nevertheless, given that numerous data indicate beneficial effects of inorganic nitrates/nitrites in patients with HFpEF and a few studies have shown neutral outcomes with NO-inducing agents, several clinical trials (NCT02980068, NCT02840799, NCT02713126) targeting this pathway are underway to resolve the controversy.

### 4.2 Soluble guanylyl cyclase stimulators and activators

sGC stimulators and activators can activate sGC to treat cardiovascular diseases independently of NO, and sGC is expected to be a promising therapeutic target for HFpEF. Treatment with sGC activators or stimulators could correct the low myocardial NO bioavailability in HFpEF by restoring downstream signals.

The DILATE-1 study, which enrolled 36 patients with LVEF>50% and a resting pulmonary artery wedge pressure (PAWP)>15 mmHg, aimed to assess the acute haemodynamic effects of riociguat in patients with diastolic heart failure. Treatment with a single oral dose of riociguat 2 mg resulted in a significant increase in stroke volume and a decrease in systolic blood pressure, indicating improvement of diastolic function, but there was no pronounced difference in PAWP [95]. SOCRATES-PRESERVED, a prospective study that included 477 subjects suffering from HFpEF with an ejection fraction ≥45%, showed that the oral sGC stimulant vericiguat failed to reduce NT-proBNP levels and left atrial volume at 12 weeks but improved patient QOL, as evidenced by the more pronounced ameliorations in physical limitations and NYHA class [96, 97]. The VITALITY-HFpEF study, a randomized multicentre trial, recruited 789 participants with LVEF≥45%, and the patients who randomly received a 24-week treatment with vericiguat did not show improvement in the Kansas City Cardiomyopathy Questionnaire-physical limitation score (KCCQ-PLS) or 6-min walking distance (6-MWD) [98]. Recently, the CAPACITY-HFpEF study, a multicentre randomized clinical trial that included 196 subjects with HF and an ejection fraction of at least 40%, indicated that participants who received praliciguat treatment for 12 weeks did not present improvement in peak VO_2_, KCCQ-PLS, or 6-MWD [99]. The reason why no favourable clinical effect on the KCCQ-PLS or 6-MWD was observed in patients in the VITALITY-HFpEF trial and CAPACITY-HFpEF trial may be that most of the patients included were NYHA class II with slight limitations in physical activity, making it difficult to evaluate changes in 6-MWD [100].

Considering that sGC stimulators and activators show an encouraging effect on QOL for patients suffering from HFpEF [96, 97], longer follow-up together with additional endpoints should be tested in further studies to justify these conclusions. For example, the DYNAMIC trial (NCT02744339), which includes 118 HFpEF participants and is designed to assess the safety and efficacy of riociguat for 26 weeks, will provide information about the effect of riociguat on cardiac output and the secondary endpoints of haemodynamic parameters.

### 4.3 Phosphodiesterase-5 inhibitors

Regulation of the intracellular concentration of cGMP mainly depends on the balance between synthesis by GC and hydrolysis by PDEs. By inhibiting the phosphodiesterase-mediated degradation of cGMP, PDE5is increase the level of intracellular cGMP, thus helping to attenuate the NO-sGC signalling deficiency.

In 2011, Guazzi M *et al.* [101] first proposed human evidence that treatment with PDE5i sildenafil for 1 year could improve LV diastolic dysfunction and exercise performance in patients with HF. In the same year, 44 HFpEF patients with ejection fraction ≥50% were recruited for a 1-year study, in which those participants assigned to the 50 mg sildenafil group displayed improved pulmonary artery pressure and ventricular function [102]. Prospectively collected data from the European COMPERA registry of 226 patients with pulmonary hypertension (PH)-HFpEF who were mainly treated with a PDE5i indicated that these patients had an improved 6-MWD, NYHA functional class, and plasma NT-proBNP level with PDE5i administration [103].

However, another clinical trial that included 52 HFpEF patients with PH and LVEF≥45% showed that compared with placebo, treatment with 60 mg sildenafil for 12 weeks failed to significantly reduce PAP or PAWP, increase cardiac output or peak VO_2_, or improve QOL in patients with HFpEF [104, 105]. Consistent with this study, the RELAX trial enrolled 216 stable HF outpatients with an ejection fraction ≥50% and found that the primary endpoints of peak VO_2_ and 6-MWD did not significantly differ between the placebo group and the sildenafil group, meaning that therapy with sildenafil for 24 weeks failed to obviously affect exercise ability or change clinical status [106]. In the RELAX trial, there was no significant difference in plasma concentrations of cGMP across the groups, allowing investigators to propose that the unsatisfactory outcomes may be associated with the inability of sildenafil to fully enhance cGMP. Wang *et al.* [107] analysed the targeted metabolomic profiling in the RELAX trial and concluded that the short-chain dicarboxyacylcarnitine metabolites from sildenafil treatment were involved in the mitochondrial dysfunction and endoplasmic reticulum stress in patients with HFpEF, which could probably explain the negative results in the RELAX trial. Another possible explanation is that sildenafil is related to an increase in neurohormone levels and renal dysfunction [108].

However, whether PDE5i can benefit certain subgroups of HFpEF patients with PH remains an open question. Given that there is no consensus on the current findings, further study is warranted to provide more in-depth information regarding the impact of PDE5is on clinical outcomes and prognosis in patients diagnosed with HFpEF.

### 4.4 Angiotensin receptor neprilysin inhibitors

The angiotensin receptor neprilysin inhibitor (ARNI) combines two different drugs—a neprilysin inhibitor and an angiotensin receptor blocker—where the neprilysin inhibitor can restore cGMP-PKG signalling in the cardiomyocytes of HFpEF patients by activating natriuretic peptide instead of the NO signalling pathway. We will discuss this class of medication therapy in HFpEF here because of its potential synergy in activating the cGMP-PKG pathway.

The PARAMOUNT trial enrolled 307 participants with an ejection fraction ≥45% and elevated NT-proBNP levels greater than 400 pg/mL and compared the primary endpoint of NT-proBNP levels between the sacubitril-valsartan group and the valsartan group. This randomized, parallel-group, double-blind, multicentre trial provided evidence that patients taking sacubitril-valsartan had lower NT-proBNP levels and better cardiac performance than those taking valsartan [109]. This study was con-sidered the foundation for the Prospective Comparison of ARNI with ARB Global Outcomes in HF With Preserved Ejection Fraction (PARAGON-HF) trial, which evaluated the quality and safety of sacubitril-valsartan compared to valsartan and their effects on morbidity and mortality in 4,822 patients suffering from HFpEF with LVEF≥45%. Over a median follow-up of 35 months, the participants randomized to receive sacubitril/valsartan therapy failed to show a marked improvement in the primary composite outcome of cardiovascular death and hospitalization for HF compared with those who received treatment with valsartan only [110]. Given that the primary endpoint was similar in the two groups, each of the secondary endpoints and subgroup analyses must be considered. Among 12 prespecified subgroups, greater benefits were noted in subjects with lower LVEF (45% to 57%) and female patients [110]. Furthermore, as recently presented, the subsequent post hoc analyses of PARAGON-HF documented that the absolute risk reductions in HF hospitalization in the sacubitril-valsartan group were more pronounced in females than in males [111], and the absolute risk reductions in primary events were more prominent in patients who were recently hospitalized within 30 days [112]. These findings suggested that population characteristics, such as sex, might affect the responses to different therapies and that such possible benefits might affect only selected subgroups due to heterogeneity.

Briefly, many new studies (NCT04687111, NCT03928158, NCT05089539) are expected to re-evaluate the efficacy and safety of ARNI therapy in patients suffering from HFpEF. For instance, the multicentre, randomized, double-blind, controlled study PARAGLIDE-HF (NCT03988634), which is designed to assess the safety and tolerability of sacubitril/valsartan and its effect on changes in NT-proBNP levels in HFpEF patients, is currently recruiting.

### 4.5 Sodium-dependent glucose transporter 2 inhibitors

Sodium-dependent glucose transporter 2 inhibitors (SGLT2is) were initially used as hypoglycaemic drugs for diabetic subjects with HF, and they were found to reduce the risk of cardiovascular events [113-116]. Further studies demonstrated that SGLT2is improved the clinical outcome of patients with HFrEF and that their cardiovascular protective effect was independent of glycaemic control [113, 117-119]. Given the excellent performance of SGLT2is in HFrEF patients, they were evaluated for use in patients with HFpEF.

In the SOLOIST-WHF study, sotagliflozin was found to reduce the risk of the composite endpoint of cardiovascular death, hospitalization for HF, and emergency department visits in diabetic patients with LVEF>50% [120]. The EMPEROR-Preserved study, which enrolled 5,988 patients with LVEF >40%, showed that patients treated with empagliflozin had an approximately 21% lower risk of the primary composite endpoint event (first HF hospitalization or cardiovascular death) and had a 27% lower risk of hospitalization for HF than those in the placebo group [11]. This benefit appeared consistent across prespecified ejection fraction subgroups and was observed in patients with and without diabetes [11]. In addition, several large randomized controlled trials have confirmed the benefits of SGLT2is in improving the symptoms and QOL in HFpEF. The EMPEROR-Preserved trial concurrently reported that treatment with empagliflozin for 12 weeks significantly improved health status and QOL in patients with HFpEF and that the effect lasted for at least 52 weeks according to the KCCQ [121]. In the CHIEF-HF trial, 476 HF participants (59% HFpEF) were randomized to the canagliflozin or placebo group. The findings indicated that patients could benefit from 2 weeks of treatment with canagliflozin regardless of ejection fraction and the presence or absence of diabetes, meaning that the HF symptoms and QOL were improved in the SGLT2i group [122]. The PRESERVED-HF trial recruited a total of 324 patients with an ejection fraction >45%, and this study showed that dapagliflozin treatment for 12 weeks could significantly improve HF symptoms, physical activity, and functional limitations in patients with HFpEF both with and without diabetes [12].

SGLT2is have been confirmed to improve the prognosis of HFpEF, but the specific mechanisms have yet to be identified, and their pharmacological effects are currently limited to preclinical research. Numerous experimental studies have shown that SGLT2is can inhibit myocardial inflammation and oxidative stress and improve endothelial function, thereby correcting the pathological suppression of the NO-cGMP-PKG pathway and its downstream targets. The activation effect of SGLT2is on the NO-sGC-cGMP-PKG pathway is supported by further experimental evidence from studies involving a nondiabetic pig model of HFpEF established by injection of deoxycorticosterone acetate (DOCA) and Ang II combined with Western diet feeding for 18 weeks and a mouse HFpEF model of spontaneous type 2 diabetes (db/db mouse) [39, 123]. The SGLT2i-induced increase in PKG activity is usually accompanied by enhanced phosphorylation of myofilament regulatory proteins such as titin and troponin I (TnI), thereby reducing the passive stiffness of cardiomyocytes [51, 124]. Together, these findings reveal important molecular mechanisms that provide a rationale for clinical studies of SGLT2is in HFpEF.

Based on the publication of the above clinical research results, in February 2022, empagliflozin was approved by the Food and Drug Administration (FDA) for the new indication of reducing the risk of cardiovascular death and hospitalization in patients with HF regardless of ejection fraction. The 2022 AHA/ACC/HFSA Guideline for the Management of Heart Failure pointed out that SGLT2is may contribute to reducing the rates of HF hospitalization and cardiovascular mortality for patients with HFpEF and that SGLT2is firstly received the recommendation for the treatment of HFpEF [125, 126]. The use of SGLT2i therapy in HFpEF has very optimistic prospects, and we thus expect the announcement of more clinical research results.

### 4.6 Other drugs targeting the NO signalling pathway

Utilizing a mouse model of hypertrophy induced by neurohormonal or haemodynamic stresses, investigators showed that activation of β_3_AR mitigated myocardial hypertrophy and fibrosis in mice subjected to isoproterenol or angiotensin II stimulation in a NOS-mediated manner [127]. This study favours the possibility of stimulating β_3_AR with the selective agonist mirabegron as a therapeutic alternative in HFpEF, and two related clinical studies are currently ongoing (NCT02775539, NCT02599480).

In an experimental rat model, treatment with the eNOS enhancer AVE3085 led to increased production of NO and a significant improvement in diastolic dysfunction [73]. The results of this research encourage us to include eNOS activators as a future therapeutic option for the treatment of HFpEF. Although the efficacy of eNOS activators has been shown in preclinical studies, clinical trials to evaluate their safety and effectiveness are still pending.

## 5. Conclusions

At present, the underlying pathophysiological mechanism and aetiology of HFpEF are not fully understood, making drug development challenging. There are still some major obstacles in adopting the interventional approaches used in preclinical animal models in clinical practice, as some of these models do not precisely reproduce the diversity and heterogeneity of human HFpEF. Knowledge of the NO-cGMP-PKG axis from abundant animal HFpEF models and human HFpEF heart tissue samples not only improves our understanding of the aetiology and pathophysiology of HFpEF but also drives extensive efforts towards identifying novel therapeutic strategies targeting the NO-cGMP-PKG signalling pathway.

**Table 2:** Ongoing or unpublished clinical trials of regulation of NO-cGMP-PKG axis in HFpEF.

study name	intervention	setting	study size	identifier ID	primary endpoint
*Inorganic nitrates/nitrites*					
PMED	oral inorganic nitrate	EF ≥40%	120	NCT02980068	nitrate/nitrite level, microbiome
INABLE-Training	oral inorganic nitrite	EF ≥50%	100	NCT02713126	peak VO2
KNO3CK OUT HFPEF	oral inorganic nitrate	EF>50%	76	NCT02840799	exercise capacity, peak VO_2_
MPMA	oral inorganic nitrate	EF ≥50%	53	NCT04913805	submaximal exercise endurance
PH-HFPEF	oral inorganic nitrite	EF ≥40%	26	NCT03015402	mPAP
*Soluble guanylyl cyclase stimulators and activators*					
DYNAMIC	riociguat	EF ≥50%	118	NCT02744339	cardiac output
*Angiotensin receptor neprilysin inhibiton*					
PARAGLIDE-HF	sacubitril/valsartan	EF >40%	800	NCT03988634	NT-proBNP
PARABLE	sacubitril/valsartan	EF ≥50%	250	NCT04687111	left atrial volume index
LCZ696 in Advanced LV Hypertrophy and HFpEF	sacubitril/valsartan	EF >50%	60	NCT03928158	6-MWD
ARNICFH	sacubitril/valsartan	EF ≥50%	60	NCT05089539	ECV
CNEPi	sacubitril/valsartan	EF ≥45%	40	NCT03506412	circulating neprilysin levels
ARNIMEMS-HFpEF	sacubitril/valsartan	EF >45%	14	NCT04753112	mPAP
*Sodium-dependent glucose transporters 2 inhibitor*					
DELIVER	dapagliflozin	EF>40%	6263	NCT03619213	CV death, hospitalization for HF, urgent HF visit
Empagliflozin in HFpEF and T2DM	empagliflozin	EF≥ 50%	100	NCT03753087	6MWD
CARDIA-STIFF	dapagliflozin	EF≥ 50%	62	NCT04739215	LV stiffness constant (S+) at the peak of exercise
The SAK HFpEF Trial	empagliflozin/potassium nitrate	EF≥ 50%	53	NCT05138575	Submaximal Exercise Endurance
Dapagliflozin Effects in HFpEF	dapagliflozin	F≥ 50%	51	NCT04730947	PCWP
STADIA-HFpEF	dapagliflozin	EF≥ 50%	26	NCT04475042	LV e'
*Other drugs targeting NO signaling pathway*					
Beta3_LVH	mirabegron	EF ≥50%	296	NCT02599480	LVMI and diastolic function
SPHERE-HF	mirabegron	--	80	NCT02775539	PVR

VO_2_: oxygen consumption, mPAP: mean pulmonary artery pressure, 6-MWD: 6-min walking distance, NT-proBNP: N-terminal pro-B-type natriuretic peptide, ECV: extracellular volume, LVMI: left ventricular mass index, PVR: pulmonary vascular resistance, PCWP: pulmonary capillary wedge pressure

The evidence of the benefits of pharmacological treatment in specific populations from subgroup analysis of the PARAGON-HF trial and (CHARM)-Preserved study [128] could encourage interventions targeting the NO-cGMP-PKG pathway and support future efforts to characterize and divide patients according to phenotype from an aetiologic perspective, which will allow tailored therapy with specific, well-defined patient cohorts to more precisely improve outcomes.

Currently, therapy with pharmacological treatment to regulate the NO-cGMP-PKG axis is being further studied in a multitude of clinical trials (Table 2), and the publication of these research results in the future is highly anticipated.

## 6. Future perspectives

Despite the inspiring scientific evidence supporting activation of the NO-cGMP-PKG pathway in the treatment of HFpEF, most of the large randomized clinical trials that assessed the effect of agents targeting this pathway have yielded negative results. Therefore, drugs (except SGLT2i) that target NO-cGMP-PKG signalling are not recommended in current guidelines. One of the primary concerns is that these strategies may contribute to hypotension, which is a potential barrier to the application of NO donors and sGC stimulants/activators. Due to side effects such as hypotension caused by the systemic vascular action of NO, the development of heart-targeted nanoparticles coated with NO inducers to avoid adverse effects on peripheral vessels is highly anticipated. Additionally, inhibition of the NO-cGMP-PKG pathway is only part of the pathological mechanism of HFpEF. According to recently reported studies, concomitant metabolic and hypertensive stress triggers nitrosative stress, which is more likely to be one of the principal drivers in HFpEF in addition to low NO bioavailability and is a possible reason why most NO-inducing pharmacological agents have failed to date. Given the heterogeneity of HFpEF and the diversity of comorbidities, the need for the development of more drugs with different therapeutic targets is urgent. Consequently, combination with drugs that interfere with other signalling pathways may help to improve the prognosis and prolong the survival time of HFpEF patients.

SGLT2is are currently the only drugs that have clearly been proven to improve cardiovascular outcomes in HFpEF and are recommended by clinical guidelines. Limited experimental studies have shown that SGLT2is could possibly exert their effects by activating NO-cGMP-PKG signalling. However, the direct therapeutic target of SGLT2is in improving cardiac function in HFpEF patients has not been fully elucidated. Given that their benefits on cardiovascular events are independent of their hypoglycaemic effects and that the SGLT2 protein is largely absent in cardiac cells [129], it is suggested that SGLT2is may not facilitate NO-cGMP-PKG signalling activation by acting on SGLT2 in cardiac tissue. A plethora of studies have indicated that SGLT2is can directly inhibit Na^+^/H^+^ exchanger 1 (NHE1), thereby reducing the intracellular Na^+^ concentration of cardiomyocytes [130-132]. Nevertheless, there is no compelling evidence to support that SGLT2is ameliorate diastolic dysfunction in HFpEF animal models or patients by blocking NHE-1. Future basic science investigations are expected to focus on the identification of their mechanism of action and the exploration of their targets and find a bridge between SGLT2is and NO-cGMP-PKG signalling, which will also help us to further understand the pathogenesis of HFpEF.
